# Supplementary material for: Outcome of Completion Surgery after Endoscopic Submucosal Dissection in Early-Stage Colorectal Cancer Patients
Source: Cancers (Basel). 2023 Sep 9;15(18):4490. doi: 10.3390/cancers15184490 (PMC10526268; doi:10.3390/cancers15184490)
Supplement: Supplementary file 1 [file cancers-15-04490-s001.zip › Additional table S1.pdf]

Additional table S1. Histological high-risk features of pT1CRC patients in the completion surgery group.

**Histological feature**	**pT1CRC completion surgery group** (n=39)
Invasion depth	
Invasion Sm2/3 or >1000	17 (60.7)
Superficial	4 (14.3)
Not assessable	7 (25)
Not described	11
Lymphovascular invasion	
Present/suspect	20 (60.6)
Absent	13 (39.4)
Not described	6
Differentiation grade	
Poor/Mucinous	5 (14.3)
Well/Moderate	30 (85.7)
Not described	4
Budding	
High grade	7 (30.4)
Absent or low grade	12 (52.2)
Not assessable	4 (17.4)
Not described	16

CRC, colorectal cancer.
