# Supplementary material for: Outcome of Completion Surgery after Endoscopic Submucosal Dissection in Early-Stage Colorectal Cancer Patients
Source: Cancers (Basel). 2023 Sep 9;15(18):4490. doi: 10.3390/cancers15184490 (PMC10526268; doi:10.3390/cancers15184490)
Supplement: Supplementary file 1 [file cancers-15-04490-s001.zip › Additional table S2.pdf]

Additional table S2. Comparison completion TME and primary TME.

**Outcome variable**	**Odds ratio** (95% CI)	***P* value**
Lymph nodes harvested[1]	0.997 (0.434-2.287)	0.993
Stoma after surgery	1.532 (0.648-3.623)	0.331
Adverse events <90 days	1.298 (0.557-3.023)	0.546
Surgical adverse events	1.376 (0.532-3.558)	0.510
Reintervention required	1.659 (0.628-4.383)	0.307
Stoma by reintervention	2.491 (0.779-7.965)	0.124
ICU admission due to complication	0.270 (0.031-2.323)	0.233
Permanent injury	NA, no events	NA
90-day mortality	NA, no events	NA

[1] Variable was dichotomized, using 12 lymph nodes as cut-off.

TME, total mesorectal excision; CI, confidence interval; ICU, intensive care unit; NA, not applicable.
